# Radiogenomic analysis of primary breast cancer reveals [18F]-fluorodeoxglucose dynamic flux-constants are positively associated with immune pathways and outperform static uptake measures in associating with glucose metabolism

**DOI:** 10.1186/s13058-022-01529-9

**Published:** 2022-05-17

**Authors:** G. P. Ralli, R. D. Carter, D. R. McGowan, W.-C. Cheng, D. Liu, E. J. Teoh, N. Patel, F. Gleeson, A. L. Harris, S. R. Lord, F. M. Buffa, J. D. Fenwick

**Affiliations:** 1 Department of Oncology, University of Oxford, Oxford, OX3 7DQ UK; 2 Doctoral Training Centre, University of Oxford, Keble Road, Oxford, OX1 3NP UK; 3 Department of Physiology, Anatomy and Genetics, University of Oxford, Sherrington Road, Oxford, OX1 3PT UK; 4 Department of Medical Physics and Clinical Engineering, Oxford University Hospitals NHS Foundation Trust, Churchill Hospital, Oxford, OX3 7LE UK; 5 Department of Nuclear Medicine, Oxford University Hospitals NHS Foundation Trust, Churchill Hospital, Oxford, OX3 7LE UK; 6 Molecular Oncology Laboratories, Weatherall Institute of Molecular Medicine, University of Oxford, John Radcliffe Hospital, Oxford, OX3 9DS UK; 7 Institute of Systems, Molecular and Integrative Biology, University of Liverpool, Daulby Street, Liverpool, L69 3GA UK

**Keywords:** Breast cancer, FDG-PET, RNA sequencing, GSEA, Glycolysis/gluconeogenesis, Immune pathways

## Abstract

**Background:**

PET imaging of 18F-fluorodeoxygucose (FDG) is used widely for tumour staging and assessment of treatment response, but the biology associated with FDG uptake is still not fully elucidated. We therefore carried out gene set enrichment analyses (GSEA) of RNA sequencing data to find KEGG pathways associated with FDG uptake in primary breast cancers.

**Methods:**

Pre-treatment data were analysed from a window-of-opportunity study in which 30 patients underwent static and dynamic FDG-PET and tumour biopsy. Kinetic models were fitted to dynamic images, and GSEA was performed for enrichment scores reflecting Pearson and Spearman coefficients of correlations between gene expression and imaging.

**Results:**

A total of 38 pathways were associated with kinetic model flux-constants or static measures of FDG uptake, all positively. The associated pathways included glycolysis/gluconeogenesis (‘GLYC-GLUC’) which mediates FDG uptake and was associated with model flux-constants but not with static uptake measures, and 28 pathways related to immune-response or inflammation. More pathways, 32, were associated with the flux-constant *K* of the simple Patlak model than with any other imaging index. Numbers of pathways categorised as being associated with individual micro-parameters of the kinetic models were substantially fewer than numbers associated with flux-constants, and lay around levels expected by chance.

**Conclusions:**

In pre-treatment images GLYC-GLUC was associated with FDG kinetic flux-constants including Patlak *K*, but not with static uptake measures. Immune-related pathways were associated with flux-constants and static uptake. Patlak *K* was associated with more pathways than were the flux-constants of more complex kinetic models. On the basis of these results Patlak analysis of dynamic FDG-PET scans is advantageous, compared to other kinetic analyses or static imaging, in studies seeking to infer tumour-to-tumour differences in biology from differences in imaging.

*Trial registration* NCT01266486, December 24th 2010.

**Supplementary Information:**

The online version contains supplementary material available at 10.1186/s13058-022-01529-9.

## Introduction

Positron emission tomography (PET) images of the radiotracer 2-deoxy-2-[^18^F]-fluoro-D-glucose (FDG) are used widely for tumour staging and assessment of treatment response [1]. In routine practice single static FDG-PET images are collected around an hour after tracer injection. In the research setting, however, PET scanning is often carried out dynamically, collecting sequences of images in time-frames from injection onwards [2]. From these sequences kinetic measures of tumour FDG uptake are obtained by analysing tumour time-activity-curves (TACs) and arterial input functions (AIFs) which describe time-courses of tracer activity concentrations within tumours and the blood flowing into them. Here we characterise associations between tumour biology and static and kinetic measures of FDG uptake, using baseline data from a non-randomised window-of-opportunity study that investigated the effects of metformin on breast cancer metabolism [3].

In the window study, static and dynamic FDG-PET scans and tumour biopsies were obtained before and after a 13–21 day course of metformin, which is used to treat type-2 diabetes and is under investigation for repurposing as a cancer therapy [4]. This has allowed us to analyse associations in the baseline data between imaging measures and tumour biological processes as inferred from sequencing of RNA from the biopsies.

Tracer kinetics are commonly estimated using multi-compartment models [5], nonparametric models [6–8] and graphical methods such as the Patlak plot [9]. The abilities of these models to describe tumour TACs have been studied previously [8, 10–12]. Relationships between FDG uptake in PET scans and expression of tumour molecular markers and genes have also been quantified [13–18], finding several involved pathways but with little consistency. To develop the utility of FDG-PET further it is important to understand more fully the connections between FDG uptake and underlying tumour biology. Moreover, the relative strengths of associations between the biology and static imaging measures and the various kinetic measures provided by different models have not been inter-compared, despite this being a key issue for interpretation of images as markers of tumour physiology.

Here, we use gene set enrichment analysis (GSEA) [19, 20] to identify biological pathways significantly associated with FDG uptake in breast cancers. Tracer uptake is quantified via the standardised uptake value (SUV) and tumour-to-blood ratio (TBR) static measures, and by kinetic indices obtained from fits of several models to dynamic data. Common elements of pathways associated with these measures are identified, and heatmaps are constructed showing the strengths of correlations between imaging measures and genes related to the common elements.

## Methods

### Patient data

#### Patients

The metformin study was prospectively approved by NHS Oxfordshire Research Ethics Committee A and registered with the ClinicalTrials.gov identifier NCT01266486. Between May 2011 and November 2013, 41 female patients from three UK centres gave informed consent and were recruited shortly after diagnosis with primary breast cancer and before instigation of any cancer therapy. Study eligibility criteria are described elsewhere [3]. Key inclusion criteria were primary tumour diameter ≥ 2 cm, Eastern Cooperative Oncology Group performance status 0–1, and fasting or random serum glucose < 7 mmol/L. Exclusion criteria included diabetes, treatments with metformin in the last year, and estimated glomerular filtration rate ≤ 45 mL/min. All patients had a magnetic resonance imaging scan as part of their routine clinical workup.

Complete baseline imaging and RNA sequencing data were available for 31 patients but analysed for 30 (median age 50 years), one AIF of the omitted patient having a sharp discontinuity, perhaps due to movement. Table 1 summarises patient and tumour characteristics.

**Table 1:** Patient and tumour characteristics

*Patients (N)*	
Total recruited	41
With PET data available	36
With PET and mRNA sequencing data available	31
Analysed	30
*ER/HER2 status (N)*	
ER positive/negative	22/8
HER2 positive/negative	5/25
Triple negative (ER negative and HER2 negative)	8
*Tumour type (N)*	
Ductal/lobular/mixed carcinoma	24/4/2
Grade 1/2/3	1/15/14
*Characteristics of 30 patients analysed (median, range)*	
Age at study entry (years)	50 (34–67)
Tumour size on MRI scan (mm)	48 (30–118)
Body mass index	26.2 (19.6–44.9)

#### PET-CT

After fasting overnight, patients were positioned supine and CT scanned for localization and attenuation correction. Dynamic PET tumour imaging was carried out for 45 min at a single bed position, injecting FDG (3 MBq/kg, up to 400 MBq maximum) 30 s after initiating data collection and grouping data into the time-frame sequence {1 × 30 s, 12 × 5 s, 6 × 10 s, 5 × 30 s, 10 × 60 s, 6 × 300 s}. At 60 min post-injection tumours were imaged again as part of a 30 min static PET scan from skull-base to mid-thigh in which data were collected for four minutes at each of several bed positions [3].

**Fig. 1:**
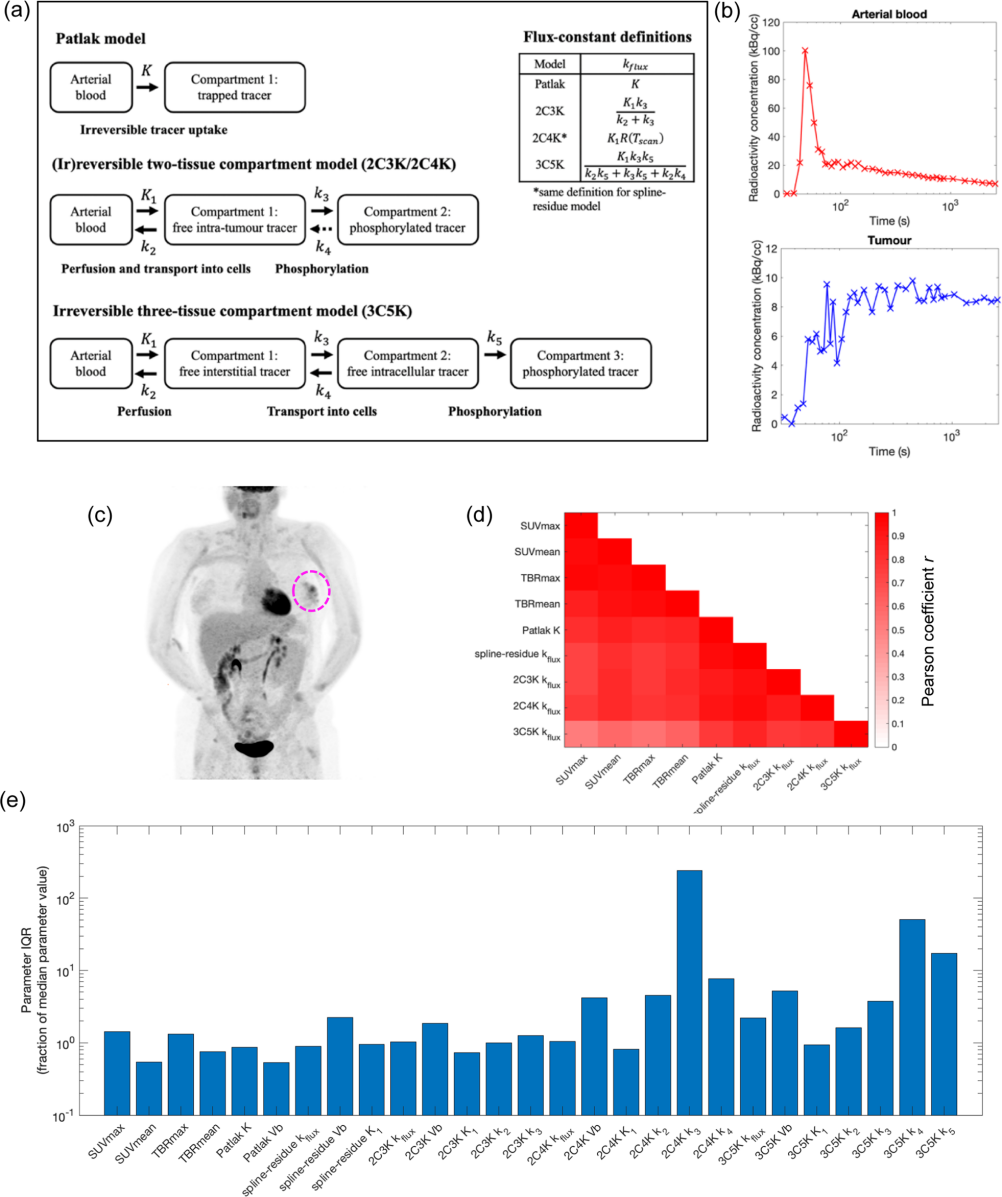
Overview of the FDG-PET imaging data and kinetic analysis. **a** Schematic diagrams of compartment models of FDG uptake. **b** Arterial and tumour time-activity-curves obtained from a patient’s dynamic FDG-PET scan. **c** Coronal maximum intensity projection through the patient’s static FDG-PET scan collected after dynamic imaging. The primary tumour lies at the centre of the dashed circle. Activity is also pronounced in the brain, heart kidneys and bladder. The static scan comprised data acquired at several bed positions whereas the dynamic scan comprised sequential images collected at one bed position. **d** Correlations between SUV, TBR and kinetic model flux-constants. **e** Interquartile ranges of model parameters

Scanning was performed using GE Discovery 690 (GE Healthcare, Chicago) and Siemens Biograph mCT-128 (Siemens Healthineers, Munich) PET-CT cameras operated in 3D-mode, both accredited for use in multicentre studies by the NCRI UK PET Core Laboratory. PET images were reconstructed on 5.5 × 5.5 × 3.3 mm^3^ voxel grids, using the FORE + FBP algorithm for the dynamic image sequence and two iterations of a 24 subset TOF-OSEM algorithm for static tumour images collected 60 min post-injection. One patient’s static FDG image is shown in Fig. 1.

#### RNA sequencing and gene expression

Tumour biopsy samples were collected 1–7 days after PET imaging. They were acquired under ultrasound guidance from primary tumour peripheries to avoid centrally-located hypoxic regions. Within one minute biopsy material was snap-frozen in liquid nitrogen prior to storage at − 80 °C. RNA sequencing was carried out for these samples at the Oxford Genomics Centre core facility of the Wellcome Trust Centre for Human Genetics. Data pre-processing was performed as previously reported [3], obtaining normalised gene expression levels as fragments per kilobase of transcript per million mapped reads (FPKM). Transcripts with zero values were removed, leaving approximately 17,000 genes whose expression values were base 2 logged.

### Image analysis

Maximum and mean SUV values, SUV_max_ and SUV_mean_, were obtained from activity concentrations within tumour contours delineated on 60-min static PET images by a nuclear medicine radiologist with eight years experience. TBR_max_ and TBR_mean_ values were calculated from the SUV measures and mean concentrations within contours (average volume 32 cm^3^) drawn on the descending aorta. Contours were transferred to corresponding dynamic PET scans, and decay-corrected 45 min-long tumour TACs and AIFs (Fig. 1) were generated using the Hermes Hybrid Viewer (Hermes Medical Solutions AB, Stockholm).

A three-exponential model [21] was least-squares fitted to the AIFs, weighting data-points according to

1 $$w_{i} = \frac{{\Delta T_{i} }}{{A_{i} }}\exp \left( { - \lambda t_{i} } \right)$$

where $\Delta {T}_{i}$, ${A}_{i}$ and ${t}_{i}$ are the duration, measured radioactivity concentration and mid-time post-injection of the *i*th time-frame, and $\lambda$ is the ^18^F decay constant [22]. The fitted AIFs provided input terms for the kinetic models, which in turn were fitted to tumour TACs.

### Tracer kinetic models

Several compartment models were investigated: standard irreversible and reversible two-tissue compartment models with 3 and 4 rate-constants (2C3K/2C4K), an irreversible three-tissue model with 5 rate-constants (3C5K), and the Patlak plot which is essentially a simple irreversible one-tissue model [3, 9, 10]. All are summarised graphically in Fig. 1. Model micro-parameters comprising the rate-constants and tumour fractional blood volume, *V*_b_, were adjusted to achieve the best fits of modelled time-courses of tumour tracer concentration to measured tumour TACs.

We also investigated a nonparametric ‘spline-residue’ kinetic model [7]

2 $$\begin{array}{*{20}c} {{\text{TAC}}\left( t \right) = K_{1} \mathop \smallint \limits_{0}^{t} {\text{AIF}}\left( s \right) R\left( {t - s} \right){\text{d}}s + V_{b} {\text{AIF}}\left( t \right)} \\ \end{array}$$

in which *K*_1_ describes blood flow into the tumour and *R*(*t*) is a residue function describing the fraction of tracer remaining within the tumour at time *t* post-injection. In this model *R*(*t*) comprises a sum of B-spline basis functions with weights adjusted to achieve the best fits of Eq. (2) to measured tumour TACs.

Flux-constants, *k*_flux_, describe the rate of tumour tracer uptake given a steady unit concentration of tracer in the blood, and provide measures of long-term FDG uptake corrected for patient-to-patient differences in AIF. For the compartment models investigated, flux-constants are given by the micro-parameter combinations shown in Fig. 1. For nonparametric models *k*_flux_ can be estimated as *K*_*1*_* R*(*T*_scan_) provided the residue function gradient approaches zero by *T*_scan_, the scan duration. Flux-constants of reversible compartment models strictly equal zero when viewed over long timescales, but operationally values of *K*_*1*_* R*(*T*_scan_) can be used for these models too, calculated from residue functions *R* corresponding to the fitted models.

### GSEA and statistics

Pearson and Spearman coefficients were calculated for correlations between logged baseline expression levels of individual genes and imaging measures, and the genes were ordered according to the correlation coefficients [19] using a random ties method for genes with identical coefficients. Ordered gene lists were parsed using the Bioconductor FGSEA simple algorithm [23] and enrichment scores were generated for pathways defined in the MSigDB-curated KEGG gene set [19, 24, 25]. These scores were compared with null-distributions obtained via 10,000 gene-wise permutations, an approach that can overestimate significance [26].

A nominal Bonferroni-adjusted p-value threshold of 0.035/N was set when identifying which pathways, from a set of N, were associated with each imaging measure. To check the true significance of the numbers of pathways identified as being associated with the various measures, we compared them with numbers of false-positive pathways in 100 synthetic datasets generated by permuting values of SUV_max_, SUV_mean_, Patlak *K* or the 2C3K model flux-constant. We also created 100 bootstrap resamples of the original baseline dataset, sampling patients with replacement. Significances of differences in the real dataset between numbers of pathways associated with any two imaging measures were determined from the distributions of differences in the bootstraps, using the paired *t*-test.

Leading-edge genes [27] shared by pathways associated with imaging measures were identified, and heatmaps were plotted of the Pearson coefficients of correlations between the imaging measures and a gene set related to the shared leading-edge genes. The Wilcoxon signed-rank test was used to assess significances of differences between distributions of Pearson coefficients obtained for different imaging measures.

Model fits to tumour TACs were inter-compared using leave-one-out cross-validation (LOOCV) and a residual sum-of-squares goodness-of-fit metric weighted according to Eq. (1). The significance of structure in model residuals was determined using the Wald-Wolfowitz runs test. Uncertainties on fitted kinetic model parameter values were calculated using a profile-likelihood method based on the weighted residual sum-of-squares, scaled by a factor in principle related to PET camera sensitivity and in practice obtained as the ratio of the degrees-of-freedom of the fit of the best model to the weighted sum-of-squares for that model fit. All reported *p*-values are two-sided.

## Results

### Imaging measures

Strengths of correlations amongst baseline imaging measures are plotted in Figs. 1 and S1 for the 30-patient cohort. All of SUV_max_, SUV_mean_, TBR_max_, TBR_mean_ and the kinetic model flux-constants were strongly inter-correlated, although 3C5K *k*_flux_ was less tightly correlated than the rest. Interquartile ranges (IQRs) of the imaging measures are also shown in Fig. 1. Some micro-parameters of the reversible two-tissue and irreversible three-tissue models had IQRs much larger than their median values, suggesting these models may have over-fitted the data.

### Glycolysis/gluconeogenesis and immune pathways were positively associated with FDG uptake flux-constants

**Fig. 2:**
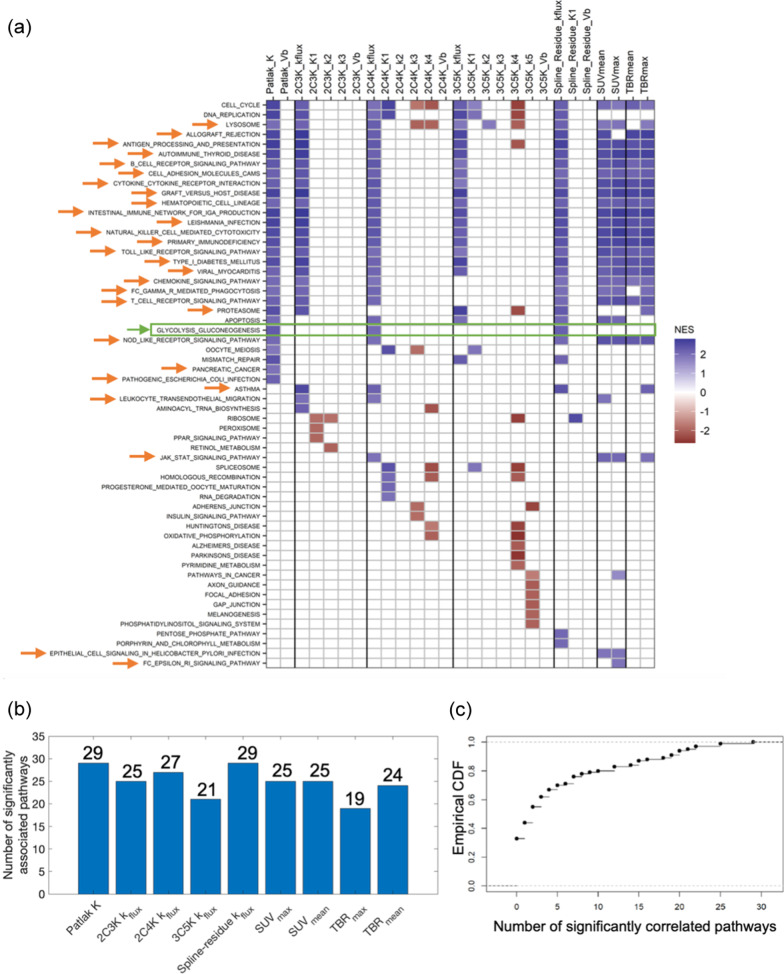
Pearson-based associations between KEGG pathways and image measures. **a** Plot showing pathways significantly associated with each imaging measure. The associated pathways are shaded by their normalised enrichment scores (NES) and GLYC-GLUC and immune-related pathways highlighted by green and orange arrows. **b** Numbers of pathways associated with model flux-constants and SUV and TBR measures. **c** Cumulative distribution function (CDF) showing numbers of pathways (false positives) associated with permuted SUV_max_ values in 100 synthetic datasets

Figure 2 shows KEGG pathways significantly associated with baseline imaging measures according to enrichment scores based on Pearson correlations. In total 38 pathways were significantly associated with the flux-constants or SUV or TBR measures, all these associations being positive. These 38 pathways included 28 related to immune response or inflammation, for example *T* and *B* cell receptor signalling pathways. They also included the glycolysis/gluconeogenesis (‘GLYC-GLUC’) pathway which involves glucose transporters and hexokinases that mediate FDG uptake [13, 28], and which was significantly associated with flux-constants of the Patlak, 2C4K and spline-residue models, but not with the SUV or TBR measures.

Both Patlak *K* and the spline-residue model flux-constant were associated with 29 pathways, more than for any other imaging measure. Fewer pathways, 21, were associated with the flux-constant of the irreversible 3C5K model than with flux-constants of other models. However, differences between numbers of pathways associated with the various model flux-constants and with the SUV and TBR measures were not significant.

For the same 0.035/N nominal Bonferroni-adjusted p-value cut-off used to categorise associations as significant in the real dataset, numbers of pathways associated with randomly permuted SUV_mean_ values had a median value of 1 (range 0–26) in 100 synthetic datasets, with ≤ 20 pathways being associated in 95% of the datasets. Comparable numbers were also obtained for randomly permuted SUV_max_, Patlak *K* and 2C3K *k*_flux_ data. The numbers of pathways significantly associated with the real unpermuted flux-constants and with SUV_max_, SUV_mean_ and TBR_max_ thus lie above levels expected by chance.

**Fig. 3:**
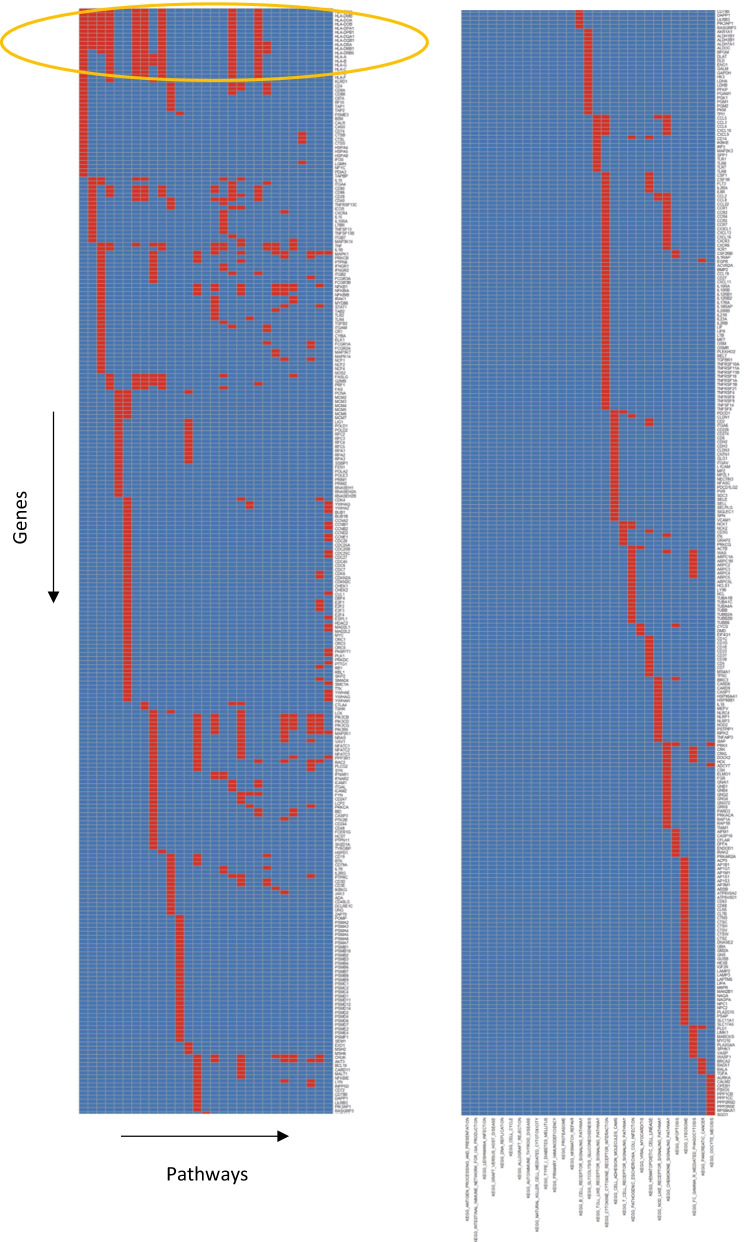
Overlaps between leading-edge genes belonging to pathways significantly associated with Patlak *K* according to Pearson-based scores. Each column shows in red genes belonging to a particular pathway. The yellow ellipse picks out 17 genes of the human leucocyte antigen group which contribute to 11 of the pathways

**Fig. 4:**
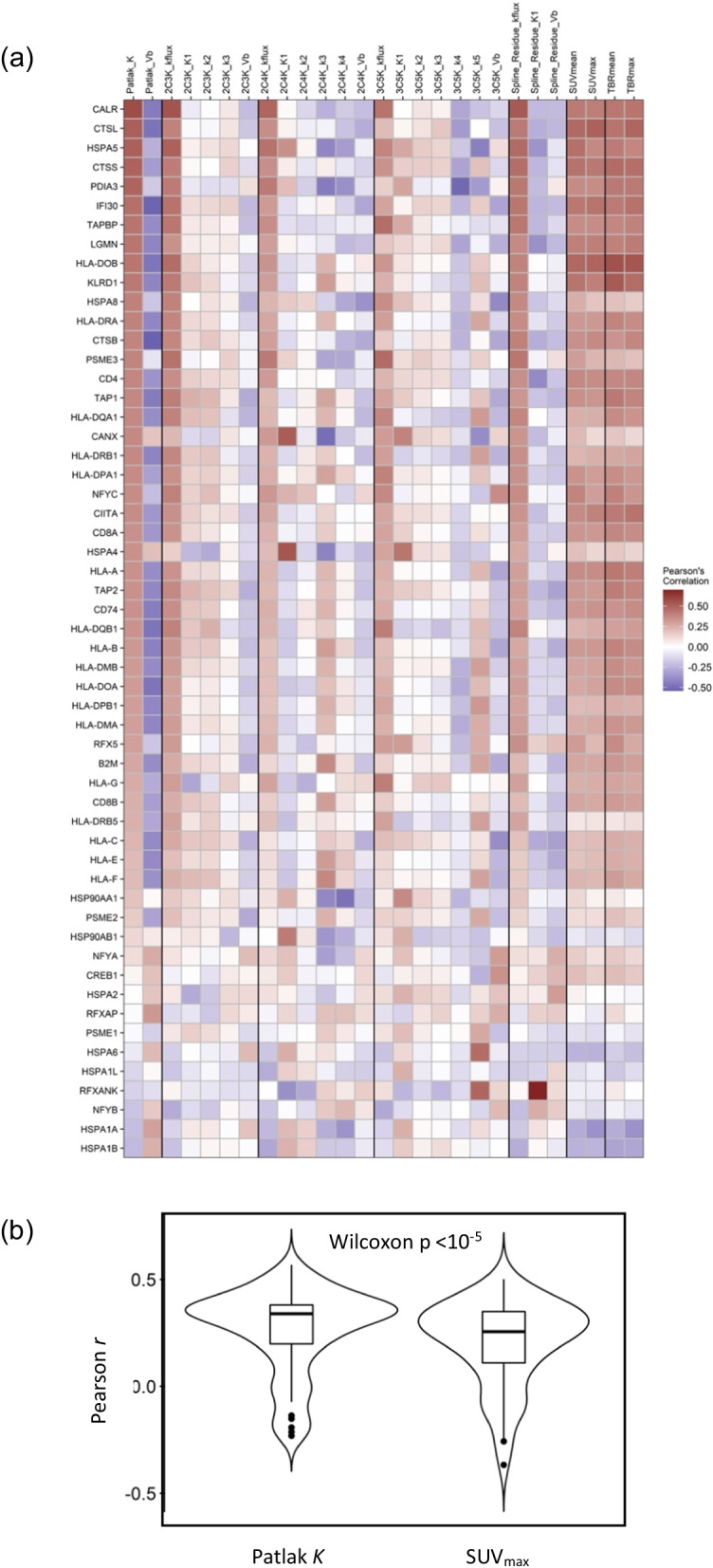
Pearson *r* coefficients of correlations between image measures and antigen presentation and processing pathway genes. **a** Heatmap of correlations between the image measures and gene expression. **b** Violin plots of *r* values for Patlak *K* and SUV_max_

Overlaps between leading-edge genes in the pathways associated with Patlak *K* are shown in Fig. 3. Eleven KEGG pathways had up to 17 genes of the human leukocyte antigen (HLA) group in common (circled), and these genes code cell surface proteins which regulate the immune system. Antigen processing and presentation was one of the eleven pathways, and had the highest Pearson-based normalised enrichment score of any pathway for correlations with Patlak *K*. Figure 4 shows a heatmap of Pearson correlations between imaging measures and the individual genes of this pathway, excluding those with low counts. Overall, the genes were correlated significantly more positively with Patlak *K* than with SUV_max_, SUV_mean_, the micro-parameters of any model, or the flux-constants of the other models apart from 2C3K.

**Fig. 5:**
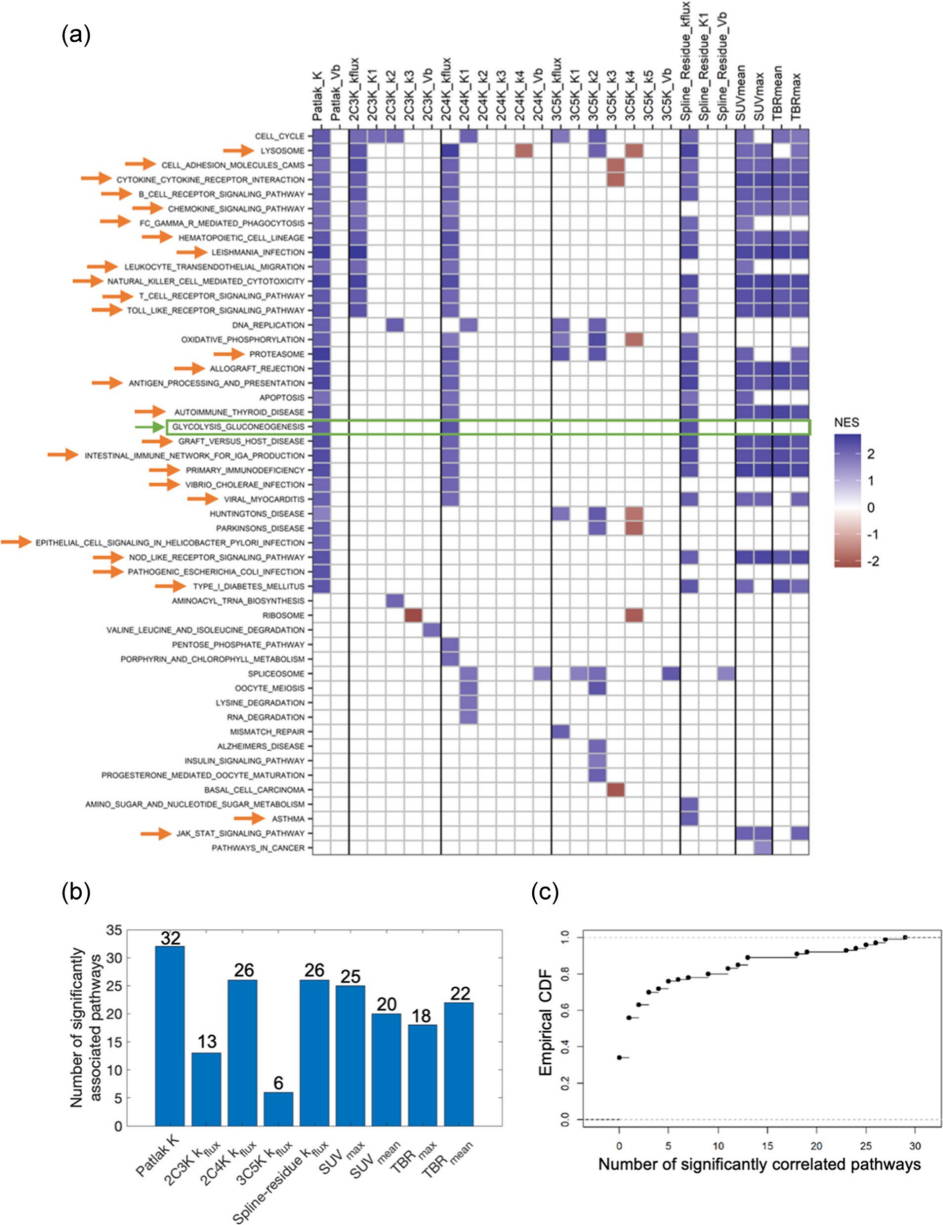
Spearman-based associations between KEGG pathways and image measures. **a** Plot showing pathways significantly associated with each imaging measure. The associated pathways are shaded by their normalised enrichment scores (NES) and GLYC-GLUC and immune-related pathways highlighted by green and orange arrows. **b** Numbers of pathways associated with model flux-constants and SUV and TBR measures. **c** Cumulative distribution function (CDF) showing numbers of pathways (false positives) associated with permuted SUV_max_ values in 100 synthetic datasets

Similar results were obtained for enrichment scores based on Spearman correlations, as shown in Fig. 5. For these scores, though, the numbers of pathways associated with Patlak *K* (32) and 2C4K *k*_*flux*_ (25) were significantly greater than the number associated with 3C5K *k*_*flux*_ (6) (*p* = 0.027 and *p* = 0.033 respectively).

### More pathways were associated with kinetic flux-constants than with micro-parameters

Between them the various kinetic models comprised 18 micro-parameters, and according to Pearson-based enrichment scores each micro-parameter was associated with fewer pathways than was the flux-constant of the model to which the micro-parameter belonged (Fig. 2). These differences were significant (*p* < 0.05) for 8 micro-parameters and borderline significant (*p* < 0.10) for another 8. In all, the micro-parameters were associated with 28 pathways, a group that included only 3 pathways related to immune-response and inflammation and excluded GLYC-GLUC. In total there were 37 negative and 13 positive associations between pathways and micro-parameters, in distinction to the exclusively positive associations seen between pathways and the flux-constants and SUV and TBR measures. One micro-parameter was associated with 13 pathways, another two with 7 pathways, and the rest with less, numbers that lie around levels expected by chance.

Results for Spearman-based enrichment scores were similar, again with more pathways being associated with flux-constants than with individual model micro-parameters, except for the 3C5K model (Fig. 5).

### Fits of the three-tissue compartment model had the largest errors

Goodness-of-fit statistics are listed in (Additional file 1: Table S1) for model fits to tumour TACs, excluding the Patlak model which is not fitted to early time-points. Of the compartment models, the irreversible two- and three-tissue models 2C3K and 3C5K had the lowest and highest median errors on LOOCV, respectively. Overall, the nonparametric spline-residue model had the lowest LOOCV error. Fits of two patients’ tumour TACs are plotted (in Additional file 1: Fig. S2) to illustrate the different descriptions of the kinetics of tumour FDG uptake provided by the various models.

### Fit uncertainties were less for *k*_flux_ than for micro-parameters of the 2C3K model

For the 2C3K model, calculated root-mean-squares of one standard deviation uncertainties obtained for fits to all tumour TACs were 11% of the mean parameter value for *k*_*flux*_ versus 16%, 26%, 28% and 21% for *K*_*1*_, *k*_*2*_, *k*_*3*_ and *V*_*b*_.

## Discussion

### Immune pathways were associated with FDG flux-constants and static uptake

Of the 38 pathways significantly associated with kinetic model flux-constants or with SUV or TBR measures according to enrichment scores based on Pearson correlations, 28 were related to immune-response or inflammation, 6 to proliferation or DNA repair and 4 to metabolism. All these associations were positive, and this was the case too for Spearman-based enrichment scores. While the pathway analysis does not determine locations or types of immune cells in detail, these positive associations suggest that enrichment of the immune-related pathways reflects a balance towards immune suppression, since higher FDG uptake is associated with a poorer prognosis for breast cancer [29]. Relatedly, SUV_max_ has been found to be positively correlated with concentrations of tumour infiltrating lymphocytes in breast tumours, and with expression of the programmed death protein 1 (PD-1) and serum levels of the chemokine CCL18 released by tumour-associated macrophages in non-small cell lung cancers [30–32]. Activation of natural killer, *B*, *T* and other immune cells is known to trigger high consumption of glucose via glycolysis [33], sparking investigations of whether tumour metabolism can be modified to improve immunity [34].

### GLYC-GLUC was associated with FDG flux-constants but not static uptake

The GLYC-GLUC pathway which mediates FDG uptake was significantly and positively associated with the flux-constants of the Patlak, 2C3K and spline-residue models according to Pearson- and Spearman-based enrichment scores, but not with SUV or TBR measures. The difference between GLYC-GLUC’s strength of association with the flux-constants versus static measures was substantial. Of the 29 pathways significantly associated with Patlak *K* according to Pearson-based scores, GLYC-GLUC was the 15^th^ most strongly associated, with an unadjusted *p*-value only 1.12 times that of the most strongly associated pathway. On the other hand, 26 pathways were significantly associated with SUV_max_, but GLYC-GLUC was only the 44th most strongly associated pathway and the unadjusted p-value of its association was 400 times that of the most significantly associated pathway. Spearman-based results were similar.

### More pathways were associated with Patlak *K* than with other measures

Across the Pearson- and Spearman-based analyses, more pathways were associated with the simple Patlak model flux-constant *K* than with any other image measure. For Pearson-based scores, differences between the numbers of pathways associated with Patlak *K* and with other flux-constants and the SUV and TBR measures were not significant; however, for Spearman-based scores significantly more pathways were associated with Patlak *K* than with the flux-constant of the most complex compartment model studied, 3C5K. This concurs with the poorer LOOCV scores obtained for fits of the 3C5K model to tumour TACs, which along with the large interquartile ranges of some 3C5K micro-parameters suggests the model over-fitted the data.

These results are consistent with the heatmap of correlations between image measures and genes of the antigen processing and presentation pathway shown in Fig. 4. Overall the genes in this map were correlated significantly more strongly with Patlak *K* than with SUV_max_, SUV_mean_ or any other flux-constant apart from that of the relatively simple 2C3K model.

Taken together, these findings suggest the simple Patlak model has advantages over the more complex kinetic models, and potentially over SUV_max_ and SUV_mean_, since more pathways were associated with Patlak *K* than with the flux-constants of other models or static uptake measures, and these pathways included GLYC-GLUC which was not associated with SUV or TBR measures. This in turn is congruent with results of Dunnwald et al.[35] who reported that survival in a cohort of patients with locally advanced breast cancer was significantly associated with kinetic but not static measures of FDG uptake.

### More pathways were associated with kinetic flux-constants or static uptake than with micro-parameters

Greater numbers of pathways were significantly associated with kinetic model flux-constants or SUV or TBR measures than with individual micro-parameters. This could be because micro-parameters represent specific FDG uptake steps (Fig. 1) whereas flux-constants are a composite measure of the whole process, or because micro-parameters are often determined less precisely than flux-constants in PET kinetic analyses [6, 10, 11, 36] as confirmed in our analysis for 2C3K, the compartment model with the lowest mean error on cross-validation.

Whereas flux-constants were significantly associated with GLYC-GLUC and many immune-related pathways, micro-parameters were associated with fewer immune-related pathways and not with GLYC-GLUC. And while all the associations were positive for flux-constants, for micro-parameters there were both positive and negative associations. These differences may reflect an absence of much real information in the associations with micro-parameters, since the numbers of associated pathways lay around levels expected by chance. Interestingly, though, the 3C5K *k*_4_ micro-parameter was associated with the oxidative phosphorylation (OXPHOS) and Huntingdon’s, Parkinson’s and Alzheimer’s Disease pathways. These pathways have several genes in common related to mitochondrial function, and previously we found post-metformin changes in the 2C3K model flux-constant of FDG uptake were associated with changes in OXPHOS expression [3].

### Study limitations

The study cohort comprised patients with a mix of tumour molecular subtypes, 22 of 30 patients being ER positive, and 25 being HER2 negative (Table 1). Regulation of signalling pathways and uptake of FDG differ between molecular subtypes [37, 38], and therefore associations between imaging measures and pathways may vary from subtype to subtype. Nevertheless, the associations reported here were significant in the heterogeneous cohort studied, in which the majority of patients (17/30) were both ER positive and HER2 negative.

Although biopsy samples used for RNAseq analysis were collected 1–7 days after PET imaging, samples were also collected 14–28 days ahead of imaging for routine diagnostic purposes. This earlier biopsy collection could lead to inflammatory changes, potentially influencing the study results. However, the relative timing of the routine biopsy collection and PET imaging corresponds well with routine scheduling of the clinical workup for breast cancer in which FDG-PET is commonly used, and so biological pathways associated with FDG images in this study are also likely to be associated with routinely collected images. And most biological pathways linked to the FDG uptake differences were immune-related.

## Conclusions

In GSEA of pre-treatment data from this breast cancer study, the GLYC-GLUC pathway which mediates FDG uptake was associated with kinetic model flux-constants, but not with SUV_mean_ or TBR_mean_. Most other KEGG pathways associated with flux-constants or with SUV_mean_ or TBR_mean_ were immune-related. The associations were positive, suggesting that in breast tumours enrichment of these pathways reflects greater immune suppression, consistent with previous findings that depletion of glucose in the tumour microenvironment by cancer cells may drive nutrient competition as a metabolic mechanism of immunosuppression [39]. More pathways were significantly associated with the Patlak flux-constant *K* than with any other index of FDG uptake. Substantially fewer pathways were associated with individual kinetic micro-parameters.

In these patients, then, tumour-to-tumour differences in biology were linked more strongly to differences in FDG uptake measured by Patlak analysis of dynamic scans than to measures obtained from other kinetic analyses or static imaging, making the Patlak approach advantageous when seeking to infer differences in biology from differences in images. And most of the biological pathways linked to the differences in FDG uptake were immune-related.

## Supplementary Information

**Additional file1: Table S1** Kinetic model goodness-of-fit statistics with best values highlighted in bold.** Fig. S1** Correlations amongst image measures in the 30-patient dataset. Correlations are shown for all the parameters of the kinetic models fitted to the data, and for SUV and TBR static measures. **Fig. S2** Examples of kinetic model fits. (**a**, **b**) Kinetic model fits to tumour TACs of two patients. (**c**, **d**) Impulse response functions (IRFs) associated with the model fits. The IRFs describe the change with time of tumour tracer concentration after injection of a unit impulse of tracer. They are given by the product of K1 and the residue function, and equal K1 at the start of imaging and approximate kflux at the end. (**e**, **f**) Fits of the Patlak model.

## Data Availability

The gene expression and imaging measure datasets which support the conclusions of this article will be made publicly available ahead of publication.
