# Supplementary material for: Radiogenomic analysis of primary breast cancer reveals [18F]-fluorodeoxglucose dynamic flux-constants are positively associated with immune pathways and outperform static uptake measures in associating with glucose metabolism
Source: Breast Cancer Res. 2022 May 17;24:34. doi: 10.1186/s13058-022-01529-9 (PMC9115966; doi:10.1186/s13058-022-01529-9)
Supplement: Supplementary file 1 — Additional file1: Table S1 Kinetic model goodness-of-fit statistics with best values highlighted in bold. Fig. S1 Correlations amongst image measures in the 30-patient dataset. Correlations are shown for all the parameters of the kinetic models fitted to the data, and for SUV and TBR static measures. Fig. S2 Examples of kinetic model fits. (a, b) Kinetic model fits to tumour TACs of two patients. (c, d) Impulse response functions (IRFs) associated with the model fits. The IRFs describe the change with time of tumour tracer concentration after injection of a unit impulse of tracer. They are given by the product of K1 and the residue function, and equal K1 at the start of imaging and approximate kflux at the end. (e, f) Fits of the Patlak model. [file 13058_2022_1529_MOESM1_ESM.docx]

**Supplementary information**

**Table S1. Kinetic model goodness-of-fit statistics with best values highlighted in bold.**

| Model | Number of fits that  pass the runs test | Median wRSS* error  from LOOCV |
| --- | --- | --- |
| Spline-residue | **30/30** | **1.59×10^5^** |
| 2C3K | 29/30 | 2.07×10^5^ |
| 2C4K | 30/30 | 2.17×10^5^ |
| 3C5K | 30/30 | 4.32×10^6^ |

*Weighted residual sum-of-squares.

**Fig. S1.** **Correlations amongst image measures in the 30-patient dataset.** Correlations are shown for all the parameters of the kinetic models fitted to the data, and for SUV and TBR static measures.

Pearson coefficient r


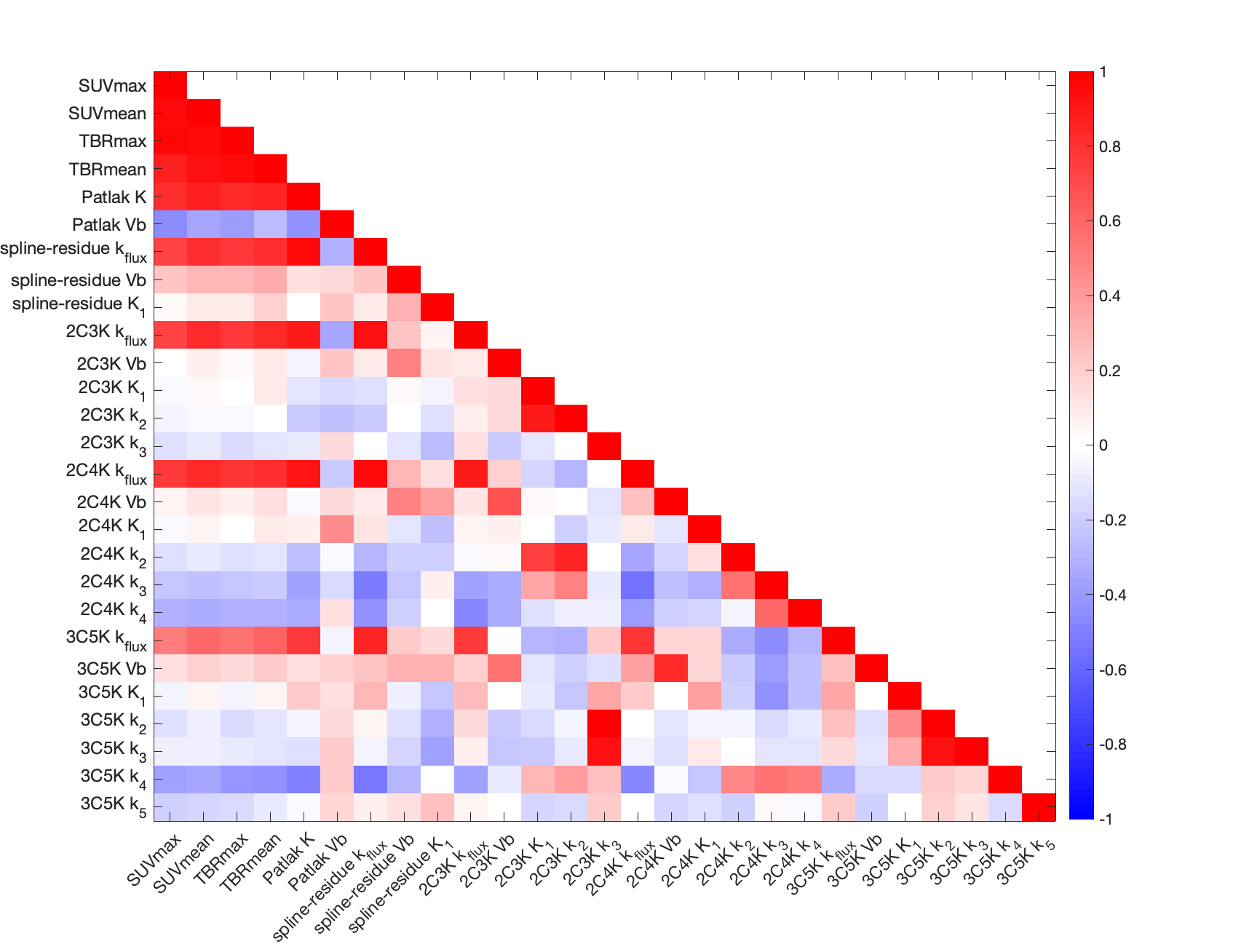


**Fig. S2. Examples of kinetic model fits.** (a,b) Kinetic model fits to tumour TACs of two patients. (c,d) Impulse response functions (IRFs) associated with the model fits. The IRFs describe the change with time of tumour tracer concentration after injection of a unit impulse of tracer. They are given by the product of *K*_1_ and the residue function, and equal *K*_1_ at the start of imaging and approximate *k_flux_* at the end. (e,f) Fits of the Patlak model.


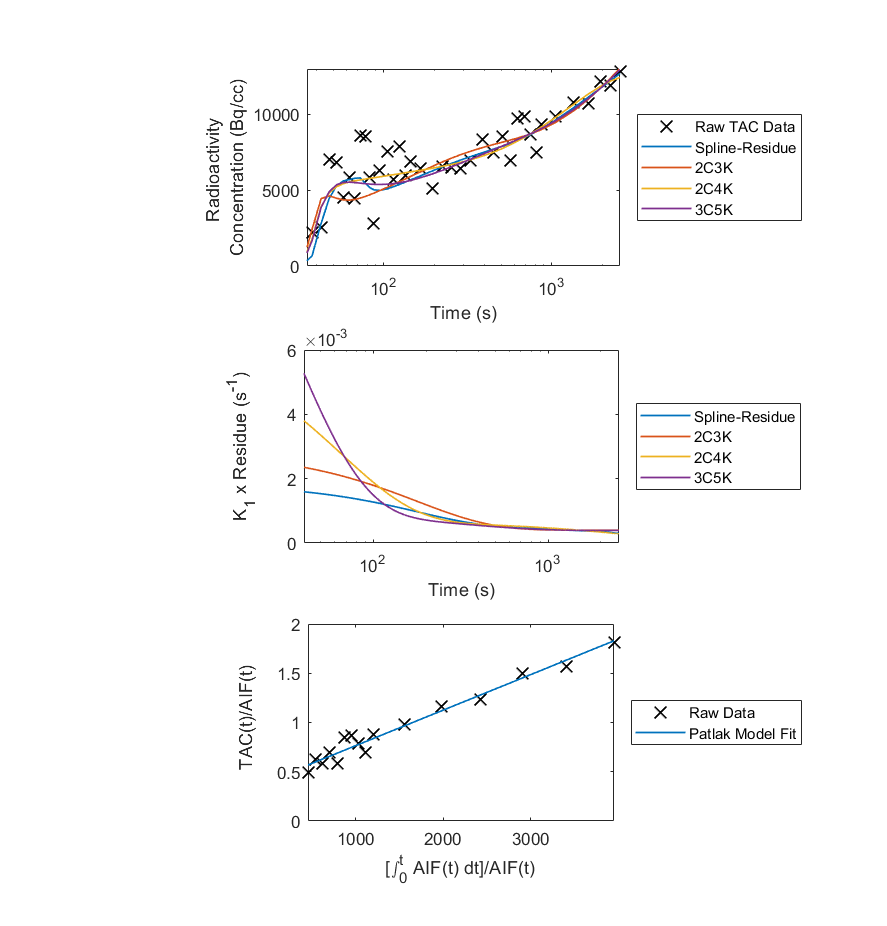

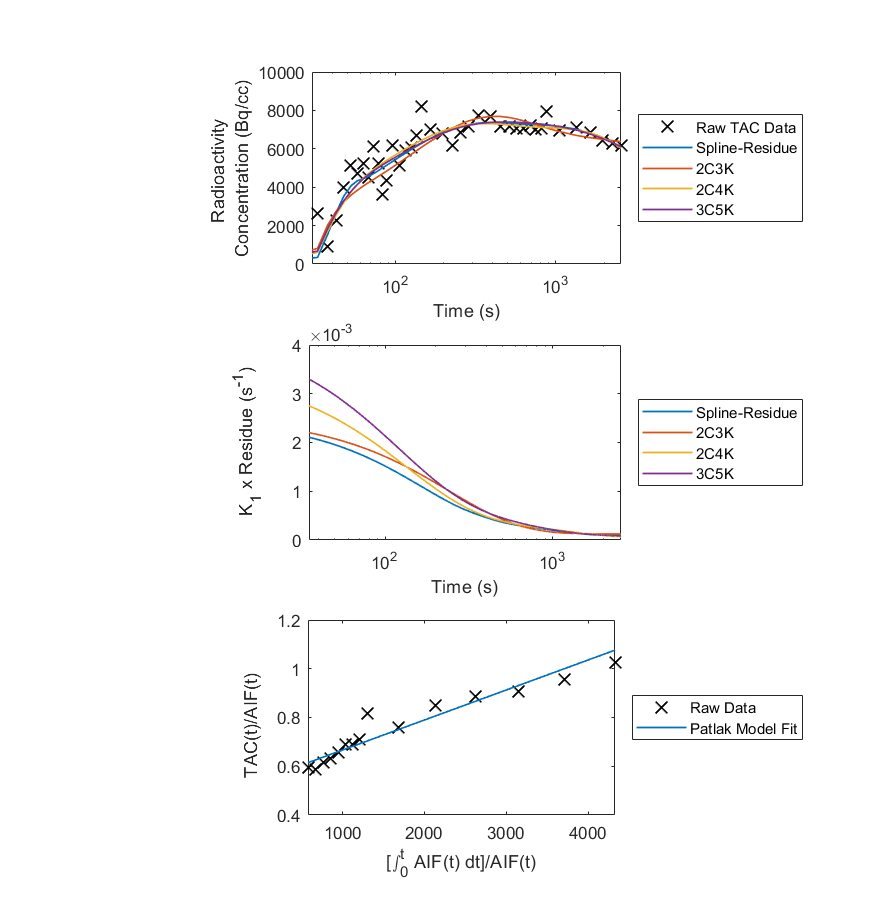


(a)

(b)

(c)

(d)

(e)

(f)
